# Guy's Hospital Reports

**Published:** 1847-04

**Authors:** 


### Art. VI.

*Guy's Hospital Reports.* Second Series. Nos. IV, V, and VI; Oct. 1844; April and Oct. 1846.—*London.*

We feel ourselves in the same predicament with these Reports, as with the Mèdico-Chirurgical Transactions previously noticed. We shall, therefore, proceed at once to our task without further ceremony.

### No. IV.

I. *Case of poisoning by opium;* by A. S. Taylor. This case is interesting from the quantity of poison taken, and the length of time that elapsed before death. The subject was a male child, aged 14 months: it must have had at least three grains of opium, and probably much more, and yet it lived for eighteen hours. No poison could be detected in the contents either of the stomach or intestines.

The narration of the case is followed by some valuable remarks on the various tests for opium, and the method of employing them. As a *trial-test,* Mr. Taylor gives a decided preference to the solution of sesquichloride of iron, which will detect the meconic acid contained in the 160th part of a grain of opium. "In order to employ it, a portion of the suspected liquid, if viscid, should be slightly diluted with water; if coloured, it should be so diluted that any change of colour, on adding the test, may be at once perceptible. The test should be saturated and neutral, or as nearly so as possible; it should be added guttatim to the suspected liquids, an equal portion of the untested solution being placed by the side of the glass, for the sake of comparing the effects. If opium be present, a dark red colour will be immediately brought out (permeconate of iron); and it will be found that this red colour is not destroyed by the addition of a few drops of a solution of corrosive sublimate. If no change of colour should be produced by the sesquichloride, and the liquid be, at the same time, in such small quantity as to admit of no further reduction in bulk by concentration, then it will be useless to seek for opium; as the quantity, if any be present, will be too small for any known process of separation."

It is a question often asked by juries,—how small a quantity of such and such a poison can you detect? To supply an answer to this question in regard to opium, Mr. Taylor performed a series of experiments with the following results.

In the tables below, the first column represents the absolute quantity of the substance detected; the second, the quantity of opium to which it is equivalent; the third, the weight of water or liquid compared with the absolute weight of the substance tested; and the fourth, the actual quantity of liquid used in the experiment:

| | 1<br>Mur. Morph. | 2<br>Opium. | 3<br>Dilution. | 4<br>Water. |
|---|---|---|---|---|
| Nitric ac. . . . | the 15th gr. | (=1·2 gr.) | 300 | 20 drops. |
| Sesquichlor. iron . | 11th | (=1·6 gr.) | 231 | 21 |
| Iodic acid . . . | 100th | (=0·18 gr.) | 1300 | 13 |

From this it is clear that iodic acid is the most delicate test for morphia, but it is open to the greatest number of objections, for an account of which we must refer to the original.

The next table shows how small a fractional quantity of meconic acid Mr. Taylor has succeeded in detecting by means of the sesquichloride of iron. The columns have the same significance as in the former one.

| 1<br>Meconic ac. | 2<br>Opium. | 3<br>Dilution. | 4<br>Water. |
|---|---|---|---|
| the 220th gr. | (= ·075 gr.) | 880 | 4 drops |
| 377th gr. | (= ·043 gr.) | 13572 | 35 |
| 570th gr. | (= ·028 gr.) | 14820 | 25 |
| 880th gr. | (= ·018 gr.) | 14080 | 16 |
| 2640th gr. | (= ·006 gr.) | 15840 | 6 |

It must be remembered that in these experiments the substances used were pure.

II. *On the action of digitalis, and its uses in diseases of the heart;* W. Munk, M.D., Physician to the Tower Hamlets Dispensary.

This is one of those performances which we hail with the greatest satisfaction; were they more numerous we should be in a better position to contend with disease, and (what is of far smaller importance, but still of some moment) to answer the frequent taunts of our homœopathic opponents.

Dr. Munk has drawn his conclusions from upwards of 400 experiments with this drug, made with care, and recorded with accuracy; and he has, we think, established some very important points.

It is well known that digitalis exerts its influence specially on two organs—the heart and the kidneys. Now it appears from the researches before us, that these results depend very much upon the preparation employed—the *tincture* affecting the heart—the *infusion* acting upon the kidneys. If it be desired to lower the action of the heart *decidedly,* as in cases of hypertrophy, the tincture should be given *alone,* in moderately full doses. If we wish to relieve the palpitations, dyspnea, &c. which form so large a portion of the distress of those who suffer from valvular disease, dilatation, &c. the tincture should be given in combination with camphor, assafœtida, musk, or other antispasmodics. In either case the patient should abstain from all exertion of mind or body. A plethoric condition is unfavorable to the action of the drug, and should be removed before its administration.

When the diuretic action is required, the *infusion* should be given in doses of from half an ounce to an ounce every six or eight hours, and the patient should take moderate exercise, and have the loins warmly clad, avoiding the production of diaphoresis.

Dr. Munk suspends its use if the pulse falls below 60, and does not persevere longer than a week, if the medicinal effects are not readily produced. With these precautions he has rarely seen any injurious effects from its employment.

III. *Remarks on the pathology of iritis;* by J. F. France, Assistant Surgeon to the Eye Infirmary.

The principal object of this paper is to establish the reality of the distinction between *syphilitic* and *arthritic* iritis, to point out the chief diagnostic marks, and the modification of treatment required.

In well-marked cases of syphilitic iritis, the first morbid changes in the membrane itself are commonly observed at or close to the margin of the pupil, where the iris is found not only to have its mobility impaired and its brilliancy tarnished, but the sharpness of that margin lost; and, as the disease advances, the natural colour of the iris, at one or more inflamed points, which appear thickened, is seen to give place to the development of a dusky reddish-brown hue. This colour, as the disease and tumefaction advance, becomes more striking, but it is sometimes obscured to a certain extent by fibrin effused into the cavity of the anterior chamber. Generally, however, there is only sufficient exudation from the free surface of the aqueous membrane to attach parts of the edge of the pupil to the capsule of the lens, and perhaps cover, more or less, its contracted aperture. In some cases, very minute dark-brown circular specks, with well-defined margins, appear in the lower segment of the cornea, and these, when present, are strongly presumptive evidences of the nature of the disease. The rusty colour of the tumefied spots on the iris Mr. France has found to depend upon a network of distended vessels ramifying over them.

In arthritic iritis the dullness of the anterior chamber is much more extensive and considerable; and, if the disease be not quickly checked, the entire circle of the pupillary margin is soon fixed by lymph to the capsule of the lens, and even the whole area of the pupil may be thickly overspread with this effusion, which presents very generally a white or yellowish-white appearance. The surface of the iris is not partially discoloured in rusty-looking spots, but has an uniform greenish or yellowish hue. In fact, in this form, the lining of the anterior chamber and the serous covering of the iris are, in common with the sclerotic, the principal seat of disease, while, in the syphilitic form, the inflammation chiefly attacks the parenchymatous structure.

In treatment of the arthritic variety, antiphlogistic measures must be more sparingly used than in the syphilitic, and the constitutional effect of mercury is not required. Conium sometimes exerts great influence over this form of disease when given in doses of five or ten grains thrice daily. An early recourse to tonics and alkalies is generally requisite.

The paper is illustrated by several well-narrated and instructive cases.

IV. *Account of a specimen of partial fracture of the neck of the thigh-bone, and of the proper source of nutrition of the head of the bone;* by J. Wilkinson King. With two plates.

V. *On paracentesis thoracis. Additional cases;* by H. M. Hughes, M.D. The whole number of cases now amounts to 25. Of these 13 have fairly recovered, 2 have at least partially recovered, and ten have ultimately died of other diseases, generally connected with that for which the operation was performed, but entirely independent of its performance.

VI—and No. VI, VIII. *Select clinical reports, with observations;* by George H. Barlow, M.A., M.D. We join these two valuable communications together, because the facts and arguments in both are directed to the establishment of the same general principles ; and we may state, *in limine,* that we have rarely met with a better specimen of the application of sound inductive reasoning to the elucidation of any point in pathology.

The first portion is occupied with the narration of two cases of fatal obstruction of the bowels. In the first case, the seat of obstruction was in the colon, the sigmoid flexure of which twisted upon itself, so as to cause two points of constriction. In the second, the jejunum was compressed by a band of false membrane, which also narrowed the cavity of a portion of ileum passing under it. In neither case was the cavity of the gut entirely closed. But, though the pathological conditions were so similar in nature, the symptoms varied in some important respects. In the first case there was little or no abdominal pain at the commencement ; in the second it was one of the earliest symptoms. In the first case there was great flatulent distension of the abdomen ; in the second there was none. In the first the urine was abundant in quantity ; in the second that secretion was almost entirely suppressed. In the first case the circulation was well maintained in the extremities, until symptoms of perforation manifested themselves ; in the second there was early collapse. Now these differences were clearly dependent upon the variation in the seat of obstruction, and accordingly, from a consideration of them and the results of other case, Dr. Barlow has grounded upon them the following rules of diagnosis in these often obscure cases :

"It appears, then, that in cases of constipation, where there is great abdominal distension, with little or no sickness till a late period of the disease, and an abundant secretion of urine, the seat of the obstruction is in the colon or rectum ; and if there be no great degree of pain or tenderness, this obstruction is probably dependent upon some mechanical cause. When, on the other hand, the abdomen appears empty, or but moderately distended—when there is early vomiting and great deficiency of urine—and, more especially, when to these symptoms there are added considerable pain, with a tendency to collapse, the affection is probably in the course of the small intestines, and, for the most part, near the pylorus ; but when the last-named symptoms are present, with the exception of deficient urine, the disease is either in the cæcum or lower part of the small intestine."

In respect of treatment, Dr. Barlow most wisely deprecates the persevering use of drastic purgatives, as being inefficient in themselves, and calculated to produce serious mischief.

"Supposing, then," he says, "in a case of constipation which has lasted several days, that it appears probable, from the distension of the abdomen, the absence of sickness, and an abundant secretion of urine, that the obstruction is situated in the lower bowels, the first step should be to exhibit some efficient but not irritating purgative, such as a full dose of calomel with half a grain of opium, followed, in a few hours, by castor-oil, which may be again repeated—or four or five grains of compound extract of colocynth, with one of extract of hyoscyamus, may be given every hour, for six or eight hours; or, should time be allowed by the absence of any very urgent symptoms, both the above means may be tried. Copious enemata should also be administered once or twice in the twenty-four hours; and where there exists the slightest suspicion of any inflammatory action, a full bleeding should be early had recourse to.

"Should no evacuation be obtained by these means, it may be well to explore the rectum with a finger or a bougie, or both, with a view to ascertaining the existence of any obstruction in that part of the bowel. The flexible tube should next be cautiously introduced, and the lower part of the bowel well washed out by copious injections of warm water, which may be drawn off by the stomach-pump. After this has been done two or three times, there will generally be an opportunity of feeling the extremity of the tube through the abdominal parietes, by which its direction and the position of the bowel may be ascertained. . . . . . . Should there be a simple distension, it is probable that the upper portion of the intestine will empty itself into the distended part; and, with a view of promoting this, a moderate purgative may again be administered, and a turpentine enema thrown up by the flexible tube. But, should no evacuation be procured in this manner, it will become probable that there is a second obstruction above the distended portion. The farther exhibition of purgatives by the mouth will then be useless, or even injurious, and our efforts should be limited to the persevering washing out of the lower bowels, and the occasional exhibition of calomel and opium; as it may happen even yet, that, by diminishing the distension, the pressure at the points may be so far diminished as to allow of the passage of the contents of the bowel."

Should, however, all these means fail, it will be advisable to open the colon in the loins.

We now come to the inquiry, how does obstruction of the upper part of the intestinal canal influence the secretion of urine? And this leads our author into a very interesting discussion, in which he attempts, and we think successfully, to prove the truth of the following proposition:

"If a sufficient quantity of water cannot be received into the small intestines, or the circuit through the portal system into the vena cava ascendens, or thence through the lungs and heart into the systemic circulation, be obstructed, or if there be extensive disorganization of the kidneys, the due secretion of urine cannot be effected."

Into the steps of his argument we have not space to enter: they consist of a series of cases, illustrating in succession the effects of obstruction in the several parts of the circuit above noticed, and the existence, in each, of an abnormal condition of the urinary secretion. Thus, in the case already alluded to, the sickness prevented the reception of a sufficient quantity of water into the system; in case 4, the ascending cava was filled with morbid deposit; in cases 5 and 6, there was obstruction to the circulation of blood through the lungs; in case 7, obstruction to the return of blood from the lungs; in case 8, obstruction to the passage of blood through the heart; and in case 9, obstruction to the passage of blood from the heart. In all these the one constant symptom was a great deficiency in the quantity of urine. The series is completed by the narration of a very remarkable case, in which, from extensive disorganization of the kidney, scarcely any solid matters were eliminated by that organ, and the patient died comatose, though a large quantity of watery fluid was passed daily. We strongly recommend these papers to the careful attention of our readers. They are illustrated by five plates.

VII. *Medical reports from the books of the clinical wards, with remarks;* by J. C. Brereton. These are interesting, and well worth reading; but do not admit of analysis.

VIII. *Report of cases of injuries to the abdomen.*

IX. *An abstract of the two half-yearly Reports of the Clinical Society, for* 1843; by E. L. Birkett, M.B.

No. V.

I. *On the pathology of phthisis;* by T. Addison, M.D., Senior Physician to the Hospital. With five coloured plates.

In this paper the author pursues the course of his former investigations, bringing forward additional evidence in support of his position, that indurations of the substance of the lungs may give rise to all the ordinary signs of tubercular phthisis, and prove fatal, without there being a single tubercle in any part of those organs. To this form of disease he gives the name of *pneumonic phthisis.*

This pneumonic phthisis may be acute; the deposits and inflamed tissue softening down and disorganizing at once, without any attempt whatever being made at induration or repair, thereby constituting one form of acute or galloping consumption. It may be *acuto-chronic;* of which there are three varieties. 1. The inflammation, though more or less acute, is slower and more insidious in its course, and manifests some attempts at repair, as indicated by various stages and degrees of induration. The induration, nevertheless, is not complete; the pulmonary tissue continues to be friable; and sooner or later, that is to say, in a few weeks or months, softens down and gives rise to excavation; most frequently by a slow ulcerative process; more rarely by an actual slough of greater or less portions of the indurated but still friable pulmonary tissue. 2. Inflammation may supervene upon or around ancient induration, leading to disorganization either of the newly-inflamed tissue, of the old induration itself, or of both at the same time. Lastly, pneumonic phthisis may be *chronic;* of which, also, there are two varieties: first, that in which old indurations undergo a slow progress of disintegration, giving rise to vomicæ; and, secondly, that very rare form of the disease, in which an insidious inflammation proceeds very slowly to convert a considerable portion of pulmonary tissue into gray induration, without any necessary excavation whatever.

*Tuberculo-pneumonic phthisis.* By this is meant a very common form of the complaint, in which, although tubercles are present, the really efficient cause of the phthisical mischief is pulmonic inflammation.

Dr. Addison distinguishes two forms of simple pulmonary tubercle, which he designates by the names *sthenic* and *asthenic:* the former being usually of a gray colour, and semi-transparent; the latter, from the very first, and however minute, of an opaque white, or boiled-rice colour, sometimes with a faint tinge of yellow. It is the sthenic variety that usually preponderates in the form of phthisis we are now considering.

"Pulmonary tubercle has its seat in the delicate filamentous tissue which forms the slight filmy parietes of the air-cells, and bears the same relation to these parietes that tubercle does to serous memorane.

"The apparent growth and increase of size of simple pulmonary tubercle depend upon changes taking place in adjacent cells, either from the development of additional tubercles, or from inflammation. The so-called enlarged tubercles, therefore, are in reality either aggregations of simple tubercles, or simple tubercles inclosed in the products of inflammation. To the former I would apply the term *compound tubercles,* which, of course, are made up of two distinct elements, viz. the abnormal product, tubercles, and the tissue of the air-cells in which that tubercle is developed. The latter element of compound tubercle, although little considered, probably plays a by no means unimportant part in some of the most serious changes observed to take place in tuberculated lungs. Compound are at all times more liable to disintegration than simple tubercles.

"When tubercles are present, and especially when numerous, in clusters, or compounded, but still quiescent, the neighbouring pulmonary cells pretty uniformly afford indication of compensatory or excessive function; the cells being more or less enlarged, as observed in pulmonary emphysema. This compensatory change probably increases the difficulty of recognizing simple tubercles in the lungs; it being rarely, if ever, possible to detect them, either by dullness of sound or percussion, or by diminished respiratory murmur, as determined by auscultation—unless, perhaps, they exist in great numbers, and in groups of considerable size.

"In general, the only physical indication worthy of the smallest confidence at a very early period is a certain degree of inequality in the respiratory murmur; it being a little more loud or puerile at certain points, or in one lung than the other.

"When tubercles are present in the pulmonary tissue, there exists a great proneness to increased vascular action, congestion, or inflammation in the lungs themselves, and their appendages, the larynx, trachea, bronchi, bronchial glands, and pleuræ. This tendency is, however, most strongly marked in the immediate vicinity of the tubercles. Unless subjected to causes calculated to aggravate such tendency to congestion or inflammation, an individual may experience little or no inconvenience from the presence of tubercles;—a pathological principle applicable to sthenic tubercles generally, whether situated in the lungs, the pleuræ, peritoneum, or arachnoid. The inflammation which supervenes upon tuberculated lungs is commonly of the low and insidious kind observed in scrofulous and cachectic constitutions; and, unless under aggravation, is rarely attended with sufficient serous exhalation to produce perfect pneumonic crepitation.

"The commencement of this inflammation, either in the pulmonary tissue itself, or in the bronchial tubes, is the ordinary commencement of tuberculo-pneumonic phthisis; and hence it is that most of the symptoms and physical signs of phthisis may manifest themselves, without evidence of a primary change in the tubercles themselves, being discoverable after death. When the pulmonic inflammation is more considerable than usual, the characteristic heat of skin often remains uninterruptedly for days together, whether accompanied by diminished, increased, or varying perspiration.

"The physical signs which become apparent as the disease advances, viz. feebleness or absence of respiratory murmur, bronchophony, tubular respiration, and dullness of sound on percussion, are more the results of this inflammation than of the extension of tubercles; and may occur from a similar cause, in pneumonic phthisis, without a tubercle ever having existed in the lung at all. These is often a well-marked relation to be observed between the degree and permanency of the characteristic pneumonic heat of skin when present, and the rapidity and extent of these local changes, as ascertained by auscultation and percussion.

"Although the red hepatization occurring in tuberculo-pneumonic phthisis very often passes quickly into softening, and the consequent formation of a cavity, nevertheless, when actual albuminous matter is thrown out, it, like that resulting from pneumonic inflammation without tubercle, usually manifests some attempts at repair; as indicated by more or less hardening and contraction of the deposit itself, and of the pulmonary tissue into which it is effused. These results of inflammation, have been very commonly, but erroneously, regarded as mere varieties of tubercular infiltration. It is the contraction of these deposits that chiefly occasions the diminished size of the lung and flattening of the ribs. Pleurisy, doubtless, very often contributes to the same result; but mere excavation of a portion of a lung has no such effect as diminishing its size. In the worst and most excavating forms of emphysema there is no shrinking of the lung.

"The attempts at repair, or induration, of the pneumonic deposits occurring in tuberculo-pneumonic phthisis are commonly very imperfect, and not durable; so that the deposits for the most part, sooner or later, undergo a second change, by which they soften down and produce excavation. This softening, however, may take place days, weeks, months, or, I believe, even years, after the original deposition. When the disease has proceeded to excavation, the natural cure of the ulcer thus produced consists in the formation of a more or less permanent lining membrane—the true cicatrix of such ulcers. Although this membrane may perhaps, now and then, remain passive and harmless for years, it most commonly happens that the cicatrization is imperfect and incomplete; the efforts at repair fail, the albuminous material, which ought to form the membrane, softens down, and with it successive portions of the pulmonary tissue furnishing it; and thus the ulceration proceeds, till, exhausted by unceasing irritation and imperfect nutrition, the patient dies."

In treating this form of phthisis, our main object should be to prevent the deposition of tubercles, by improving the general health; and when they are found to oppose the progress of inflammatory action, by moderate general and local bleeding, active and continued counter-irritation, &c., and sometimes by the use of mercury.

*Tubercular phthisis* is characterized by a preponderance of compound asthenic tubercles; and, in Dr. Addison's opinion, is incurable. His remarks upon it are most judicious; but as this is the form of phthisis with which our readers are most familiar, we shall be contented with simply directing their attention to the paper itself.

Dr. Addison's papers claim the best attention of the pathologist and practical physician.

II. *Cases in medical jurisprudence, with remarks;* by A. S. Taylor.

This communication is occupied with the subject of poisoning by prussic acid, and is full of interest. The author has, we think, conclusively shown that the *shriek*, concerning which so much has been written and said of late, is no necessary accompaniment of poisoning by this drug; that the same is true of convulsions; that, after even a large dose has been taken, there may be sufficient time and consciousness for the individual to cork and put away the bottle, and arrange the bedclothes; that the absence of odour is no proof of the absence of the poison; and that, on the other hand, the odour may often be detected when chemical analysis gives only negative results.

In regard to tests, the nitrate of silver and the prussian-blue tests, are of nearly equal value in practice, though the former is in reality the most delicate. We must quote a short passage illustrative of the way in which the silver may be used successfully as a trial-test, even when the poison is extremely diluted.

"A small quantity of the diluted liquid was placed in a watch-glass, another watch-glass, containing one drop of a solution of nitrate of silver, was inverted over it. On holding the two glasses in the warm hand for a few minutes, a fine film of cyanide of silver was deposited over the upper glass in and about the spot wetted by the nitrate, care being taken that there should be no contact of the two liquids. This experiment shows not only the extreme volatility of the poison, even when mixed with 10,000 times its weight of water, but also that the nitrate of silver is an extremely delicate trial-test, which may enable us to detect the poison, even in a liquid which has no odour. It is, however, absolutely necessary, in such a case, that we should take advantage of the volatility of the acid, and not apply the test at once to the diluted liquid."

III. *Illustrations of some of the forms of sudden death from disease, as it occurred to nineteen Individuals in the Manchester Workhouse, with remarks bearing upon the diagnosis and pathology of such cases;* by D. I. T. Francis, M.B.

This is a useful paper, and well arranged. It will repay perusal.

4. *Reports of cases of diseases of children, treated at Guy's Hospital in* 1843-4, *with remarks;* by Golding Bird, A.M., M.D.

The cases narrated include remittent fever, croup, infantile syphilis, and scarlatinal dropsy. We have only space to remark that the occurrence of the last-named affection is attributed by Dr. Bird to the retention of some of the morbid poison of scarlatina in the blood, the free action of the skin being at the same time impeded, or suspended by want of due care. We must also transcribe his account of a very simple and easy process for the detection of urea in the blood and serous fluid:

"Allow the blood to coagulate, decant the serum, and agitate it violently with its own bulk of rectified spirit; a dense deposit of albumen occurs, and the mixture may be set aside for subsequent examination, or, if time permits, this may be proceeded with immediately. For this purpose throw the whole on a filter, and evaporate the filtered fluid slowly to a drachm or two; then add to it an equal bulk of dilute nitric acid of the Pharmacopœia, and once more filter. The filtered fluid, collected in a watch-glass, may be slowly evaporated to a few drops, and, on cooling, feathers of nitrate of urea will form in the liquor. Should the crystallization be imperfect, the deposited nitrate may be redissolved in a few drops of water, the solution decanted, and once more slowly evaporated."

In the second, non-inflammatory stage of hooping-cough, Dr. Bird has found alum a most valuable remedy, diminishing the secretion and relieving the cough. The following is the formula he has generally used for children of two or three years: ℞. Aluminis, gr. xxv; Extr. conii, gr. xij; Syr. rhæad., ʒij; Aq. anethi, ℥iij. M ft. cochl. j; med. 6tâ. quâque horâ.

5. *Report of cases of Injuries to the Chest.*

### No. VI.

I. *Two cases of labour, protracted by insuperable rigidity of the os uteri, with remarks;* by John C. W. Lever, M.D.

In the first of these cases the os and part of the cervix uteri were forced off during a pain. The patient died of puerperal fever. In the second, the rigid and unyielding os was incised on each side, and the patient did well. Dr. Lever believes this to be the best method of procedure, when the obstacle will not yield to the effects of bleeding, antimony, opium, warm bath, &c. He advises the incisions to be made at the sides, and during a pain. Artificial dilatation he considers most dangerous. We agree with him that in such extreme cases it is not advisable; but we are confident, from the results of considerable experience, that when the rigidity is of a minor degree, *gentle* dilatation with the finger is a most valuable adjunct to the other means, and we have never seen any injury produced by its judicious employment.

II. *Trial for murder by poisoning with arsenic, Berkshire Lent Assizes,* 1845. *Post-mortem appearances; chemical analysis; detection of the poison as sulphuret, twenty-eight days after interment;* by Alfred S. Taylor.

III. *Case of ossification and dislocation of the crystalline lens, with remarks;* by J. F. France.

This is an account of an extremely rare morbid change, resulting from injury received twenty years before the patient was seen. The dislocation took place spontaneously after that prolonged period. The lens was analysed by Dr. Rees, and found to contain, carbonate of lime, 0·5 gr.; phosphate of lime, 0·3 gr.; animal matter, 0·2 gr.

IV. *Case of popliteal aneurism, and of ligature of the femoral artery;* by John Nottingham, M.D., Liverpool.

A needlessly elaborate record of a very ordinary case.

V. *On the pathology and treatment of fracture of the neck of the thigh-Bone*; by Bransby B. Cooper, F.R.S.

The object of this paper is to demonstrate the impossibility of osseous union of fracture within the capsule, and, consequently, the uselessness and impropriety of employing the various contrivances which have, from time to time, been proposed for this purpose. The most novel portion is the record of some analysis of the proportion of earthy matter in the neck and shafts of old bones; an abstract of which we subjoin:

AVERAGE.

| | | |
|---|---|---|
| 1. Fractured neck of the femur yielded per cent. of bone earth | 23·9 | |
| Shafts of same bones | | 50·1 |
| 2. Unfractured neck of old bone, not buried | 33·5 | |
| Shafts of same bones | | 55·5 |
| 3. Unfractured neck of unburied bone of middle-aged person | 50·1 | |
| Shaft of same bone | | 56·7 |
| 4. Unfractured neck of old bones, very dry and exhumed | 61·4 | |
| Shafts of same bones | | 64·9 |

VI. *Abstract of the two half-yearly reports of the Clinical Society for* 1844. Part I. Medical Report; by E. Lloyd Birkett, M.B. Part II. Surgical Report; by Robert Gosset, Surgical Secretary to the Clinical Society.

VII. *Two cases of extra-uterine fœtation;* by Dr. Oldham.

The first was an instance of tubal pregnancy; the second Dr. Oldham regards as an example of interstitial or parietal extra-uterine pregnancy. It presented a very curious anomaly; the right Fallopian tube having apparently received an ovum from the left ovary, and transmitted it to the artificial sac, formed in the substance of the right horn of the womb. This result was probably brought about through the medium of false membranes, which were very numerous; but some degree of uncertainty hangs over the whole case, from the fact that the ovum was not found. Death was caused in both instances from the rupture of the large vessels in the walls of the containing sac. In both the uterus was lined with decidua.

These Reports are in the hands of so many of our readers, that we the less regret being compelled, for want of room, to give so imperfect a notice of them.

---

